# Epidemiology and Short-Term Hospital Outcomes of Skiing- and Snowboarding-Related Tibial Shaft Fractures Compared With Other Mechanisms of Injury

**DOI:** 10.7759/cureus.113161

**Published:** 2026-07-22

**Authors:** Daniel Liu, Puranjay Gupta, Yiru Wang, Michael Allen, Sam Jiang, Lisa Cannada, Jamal Ahmad

**Affiliations:** 1 College of Medicine, University of Illinois Chicago, Chicago, USA; 2 Department of Neurosurgery, Emory University Hospital, Atlanta, USA; 3 Department of Orthopaedic Surgery, Novant Health, Charlotte, USA; 4 Department of Orthopaedics, University of Illinois Chicago, Chicago, USA

**Keywords:** injury severity, length of stay, mechanism of injury, national trauma data bank, resource utilization, short-term outcomes, skiing injuries, snowboarding injuries, tibial shaft fracture, trauma epidemiology

## Abstract

Background

Tibial shaft fractures are common long-bone injuries associated with significant healthcare utilization. While winter sports such as skiing and snowboarding are recognized etiologies of lower-extremity trauma, literature regarding tibial shaft fractures following such activities remains scarce. The biomechanics associated with skiing and snowboarding may differ from other traumatic mechanisms of injury (MOIs), potentially resulting in distinct injury patterns that influence hospital outcomes and resource utilization. The purpose of this study was to characterize the epidemiology of skiing- and snowboarding-related tibial shaft fractures and evaluate the influence of this MOI on short-term hospital outcomes.

Methods

This retrospective cohort study identified 12,996 patients with tibial shaft fractures from 2017 to 2018 in the American College of Surgeons National Trauma Data Bank (NTDB), using International Classification of Diseases, Tenth Revision (ICD-10) diagnostic codes, including 232 skiing/snowboarding patients and 12,764 patients injured through other mechanisms. Patients with multiple tibial shaft fracture ICD-10 diagnosis codes or subsequent encounter codes were excluded. Skiing and snowboarding injuries were compared with all other MOIs, and 1:1 propensity score matching on age, sex, comorbidities, insurance type, injury characteristics, and hospital factors was used to reduce confounding. Hospital outcomes, including time to discharge, measured with length of stay (LOS), were evaluated in the matched cohort using appropriate comparative tests and Cox proportional hazards regression with four covariate sets: (1) demographics and injury severity score (ISS), (2) fracture characteristics, (3) payment and hospital factors, and (4) all prior covariates plus selected comorbidities and acute compartment syndrome (ACS).

Results

Skiing and snowboarding were associated with a higher hazard of discharge in Models 1-3 (HRs of 1.40, 1.77, and 1.48; all p < 0.001), suggesting shorter LOS compared with patients injured through other MOIs. However, this association was no longer statistically significant in Model 4 (HR 1.08, 95% CI 0.93-1.24, p = 0.302). Longer LOS was associated with increasing age, higher ISS, open fractures, diabetes, alcohol use disorder, substance abuse, and ACS (all p < 0.001). Segmental fractures were associated with longer LOS (HR 0.82, p < 0.001), whereas spiral, oblique, and transverse fractures were associated with shorter LOS relative to comminuted fractures (all p < 0.001).

Conclusion

Skiing- and snowboarding-related tibial shaft fractures initially appeared to be associated with shorter LOS. However, after adjustment for patient, injury, and hospital characteristics, this MOI was no longer independently associated with time to discharge. While this MOI does not appear to independently drive short-term hospital outcomes, these findings suggest, but do not establish, that skiing- and snowboarding-related tibial shaft fractures represent a distinct lower-risk injury profile characterized by younger age, lower injury severity, fewer open fractures, fewer comorbidities, and more favorable discharge disposition.

## Introduction

Tibial shaft fractures are among the most common long-bone fractures and represent a significant proportion of trauma-related fractures seen in the United States [1]. Because the tibial shaft is subcutaneous along much of its length and therefore has limited protective soft-tissue coverage, such fractures are particularly vulnerable to compromised perfusion, wound complications, and infection, especially in higher-energy patterns [2,3]. Consequently, tibial shaft fractures frequently require inpatient management, during which certain pre-surgical characteristics and post-surgical events may contribute to increased hospital length of stay (LOS). LOS is a commonly used indicator of inpatient resource utilization and costs and serves as a surrogate for the cumulative burden of the treatment course, including treatment complexity, complications, and discharge disposition [4].

Injuries in winter sports, such as skiing and snowboarding (hereafter referred to as skiing), differ from other causes of tibial shaft fractures in both the forces involved and the environments in which they occur. These sports represent distinct mechanisms of injury (MOIs) that may involve high speeds and non-physiologic fall patterns, leading to complex rotational, bending, and loading forces transmitted through the lower extremity [5]. Apart from the possible differences in biomechanical forces involved, skiing injuries frequently occur in mountainous environments where pre-hospital transport and transfer to higher-level trauma centers may be required [6]. Such geographic and logistical factors may influence patterns of healthcare utilization, including interfacility transfers, trauma center referrals, and the hospital or hospital settings in which patients ultimately receive care.

Previous studies on skiing-related tibial fractures have largely focused on injury epidemiology, protective equipment, prevention strategies, and return to sport [7]. While these studies have contributed to the understanding of injury patterns and functional recovery, comparatively little attention has been directed toward hospital-level outcomes. Determining whether skiing-related tibial shaft fractures differ in patient and injury profile, LOS, discharge disposition, or inpatient complications has important clinical implications for resource allocation, discharge planning, and trauma system utilization. Furthermore, understanding whether any observed differences reflect the MOI itself or underlying differences in patient characteristics, injury severity, fracture morphology, and hospital factors may improve interpretation of outcomes and identify patient populations that require differing levels of inpatient care.

The primary objective of this study was to evaluate the association between skiing-related tibial shaft fractures and short-term hospital outcomes such as LOS, discharge disposition, and inpatient complications, using the American College of Surgeons National Trauma Data Bank (NTDB). Secondary objectives were to characterize the epidemiology of skiing-related tibial shaft fractures, including patient demographics, comorbidity burden, fracture characteristics, and hospital factors, and to compare these characteristics with tibial shaft fractures sustained through other mechanisms of injury. We hypothesized that patients who sustained tibial shaft fractures from skiing would demonstrate a significantly shorter LOS compared to patients injured through other mechanisms due to a lower comorbidity burden, increased physical activity, and fewer risk factors associated with complications.

## Materials and methods

Patient selection and study cohorts

This retrospective cohort study identified patients with tibial shaft fractures (ICD-10-CM S82.2) recorded in the NTDB from 2017 to 2018. Patients with multiple tibial shaft fracture ICD-10 diagnosis codes or subsequent encounter codes were excluded. Entries with a skiing or snowboarding MOI (ICD-10-CM V00.32 and V00.31, respectively) were grouped into the skier cohort, and all other MOIs constituted the non-skier cohort (hereafter referred to as skiing or skiers and non-skiers, respectively, or skiing MOI and non-skiing MOI, respectively). 

Variables and outcomes

The primary outcome was time to discharge, measured by hospital LOS and defined as the time from hospital admission to discharge. Secondary outcomes included discharge disposition and inpatient complications. Patient-level covariates included age, sex, race, ethnicity, insurance type, selected comorbidities present in both cohorts, injury severity score (ISS), fracture characteristics (fracture pattern, displacement type, laterality, and open versus closed injury) based on ICD-10 coding, hospital characteristics (teaching status, ownership, size, and state trauma level), and a diagnosis of acute compartment syndrome (ACS). These variables were compared between the skiing and non-skiing cohorts to characterize the epidemiology of tibial shaft fractures by MOI, including differences in patient demographics, comorbidity burden, fracture characteristics, and hospital factors. Operative treatment was evaluated descriptively but was not included in comparative or multivariable analyses because of inconsistent procedural coding within the NTDB.

Missing data

Missing covariate data were addressed using multiple imputation by chained equations (MICE) using predictive mean matching with five imputations (m = 5) and a random seed of 123. Variables included in the imputation model were age, demographic characteristics, fracture characteristics, comorbidities, hospital characteristics, ACS status, operative treatment, and exposure status (skiing vs non-skiing). Multiple imputation was performed to preserve sample size and reduce potential bias associated with complete-case analysis. The completed dataset was subsequently used for propensity score estimation and multivariable analyses.

Propensity score matching

To reduce confounding between the skiing and non-skiing cohorts, 1:1 propensity score matching was performed using logistic regression with skiing MOI as the dependent variable. Covariates included age, sex, race, ethnicity, insurance type, ISS, fracture pattern, displacement, laterality, open versus closed injury, baseline comorbidities, hospital teaching status, ownership, size, trauma center level, and ACS status. Nearest-neighbor matching without replacement was performed using the logit of the propensity score with a caliper width of 0.2. Covariate balance before and after matching was assessed using standardized mean differences.

Statistical analysis

Baseline characteristics and outcomes were compared in both the unmatched and propensity-matched cohorts. In the unmatched sample, continuous variables were compared using independent-samples t-tests and categorical variables using Pearson χ² tests; in the matched sample, paired t-tests and McNemar tests were used, respectively.

Time to hospital discharge was analyzed using Cox proportional hazards regression with LOS as the outcome and skiing MOI as the primary exposure. Model 1 adjusted for demographic and injury severity variables (age, sex, race, ethnicity, and ISS). Model 2 adjusted for fracture characteristics (fracture pattern, displacement, laterality, and open versus closed injury). Model 3 adjusted for hospital and payment factors (insurance type, hospital teaching status, ownership, size, and trauma center level). Model 4 included all covariates from Models 1 to 3 together with baseline comorbidities (diabetes, hypertension, smoking, alcohol use disorder, substance use disorder, and COPD) and ACS status. Results are reported as hazard ratios (HRs) with 95% confidence intervals (CIs).

The proportional hazards assumption was evaluated for all Cox models. Multicollinearity was assessed using variance inflation factors (VIFs), with a prespecified threshold of VIF >4. No covariate exceeded this threshold, and all variables were retained in the final models. All statistical tests were two-sided with α =0.05 and were performed using RStudio (Posit PBC, Boston, MA, USA).

## Results

A total of 12,996 patients with tibial shaft fractures met the inclusion criteria, including 232 (1.8%) in the skier cohort and 12,764 (98.2%) in the non-skier cohort. Baseline patient, injury, and hospital characteristics are summarized in Table 1. Both cohorts were predominantly male, with similar proportions of male patients in the skier and non-skier groups (68% vs 67%, respectively). Skiers were younger (36 ± 18 vs 39 ± 21 years, p = 0.013) and more frequently White (90% vs 72%, p < 0.001). Compared with non-skiers, skiers represented a cohort with a healthier baseline, with significantly lower rates of diabetes, hypertension, smoking, alcohol use disorder, substance use disorder, and COPD (all p < 0.05). ISS in skiers was also lower compared to non-skiers (5 ± 2 vs 10 ± 9, p < 0.001) (Table 1).

**Table 1:** Unmatched and propensity-matched baseline patient, injury, and hospital characteristics in the skier and non-skier cohorts. Baseline demographic, injury, comorbidity, insurance, and hospital characteristics for patients with tibial shaft fractures, comparing skiing-related injuries with other MOIs in unmatched and propensity score-matched cohorts. Continuous variables are presented as mean ± SD and categorical variables as number (%). p-Values compare skiing and non-skiing cohorts within unmatched and matched samples. ISS, injury severity score; ACS, acute compartment syndrome; COPD, chronic obstructive pulmonary disease; SD, standard deviation; MOI, mechanism of injury. ^a^Mean ± SD; n (%). ^b^Welch two-sample t-test; Pearson's chi-squared test. *p < 0.05; **p < 0.01; ***p < 0.001.

Characteristic	Unmatched				Matched			
	Skier (unmatched), N = 232^a^	Non-skier (unmatched), N = 12,764^a^	p-Value^b^	Statistic	Skier (matched), N = 194^a^	Non-skier (matched), N = 194^a^	p-Value^b^	Statistic
Demographics								
Age (years)	36 ± 18	39 ± 21	0.013*	-2.50	36 ± 19	36 ± 21	>0.9	0.11
Sex								
Female	74 (32%)	4,185 (33%)	0.8	0.08	59 (30%)	57 (29%)	>0.9	0.05
Male	158 (68%)	8,579 (67%)			135 (70%)	137 (71%)		
Race								
Asian	6 (2.6%)	205 (1.6%)	<0.001***	47.67	6 (3.1%)	5 (2.6%)	>0.9	0.28
Black	1 (0.4%)	1,873 (15%)			1 (0.5%)	1 (0.5%)		
Other	17 (7.3%)	1,528 (12%)			13 (6.7%)	11 (5.7%)		
White	208 (90%)	9,158 (72%)			174 (90%)	177 (91%)		
Ethnicity								
Hispanic or Latino	14 (6.2%)	1,592 (13%)	0.003**	9.47	10 (5.2%)	11 (5.7%)	>0.9	0.05
Not Hispanic or Latino	212 (94%)	10,515 (87%)			184 (95%)	183 (94%)		
Insurance								
Insurance type								
Medicare/Medicaid	26 (11%)	4,411 (35%)	<0.001***	105.59	26 (13%)	24 (12%)	0.8	0.44
Other	24 (10%)	2,646 (21%)			21 (11%)	25 (13%)		
Private/Commercial	182 (78%)	5,707 (45%)			147 (76%)	145 (75%)		
Fracture characteristics								
Fracture pattern								
Comminuted	94 (41%)	5,420 (42%)	<0.001***	27.82	73 (38%)	77 (40%)	>0.9	1.51
Oblique	31 (13%)	1,231 (9.6%)			28 (14%)	31 (16%)		
Other	4 (1.7%)	548 (4.3%)			3 (1.5%)	4 (2.1%)		
Segmental	3 (1.3%)	397 (3.1%)			3 (1.5%)	3 (1.5%)		
Spiral	41 (18%)	1,281 (10%)			34 (18%)	28 (14%)		
Transverse	23 (9.9%)	1,056 (8.3%)			19 (9.8%)	15 (7.7%)		
Unspecified	36 (16%)	2,831 (22%)			34 (18%)	36 (19%)		
Displacement								
Displaced	184 (79%)	8,695 (68%)	0.001***	13.18	150 (77%)	149 (77%)	0.8	0.45
Nondisplaced	8 (3.4%)	690 (5.4%)			7 (3.6%)	5 (2.6%)		
Unspecified	40 (17%)	3,379 (26%)			37 (19%)	40 (21%)		
Laterality								
Left	120 (52%)	5,901 (46%)	0.077	5.13	96 (49%)	94 (48%)	0.8	0.35
Right	107 (46%)	6,257 (49%)			93 (48%)	93 (48%)		
Unspecified	5 (2.2%)	606 (4.7%)			5 (2.6%)	7 (3.6%)		
Open vs Closed								
Closed	204 (88%)	8,373 (66%)	<0.001***	50.64	167 (86%)	169 (87%)	0.9	0.09
Open	28 (12%)	4,391 (34%)			27 (14%)	25 (13%)		
ISS	5 ± 2	10 ± 9	<0.001***	-31.318	5.11 ± 2.30	5.08 ± 2.19	0.9	0.16
ACS	3 (1.3%)	104 (0.8%)	0.7	0.64	1 (0.5%)	3 (1.5%)	0.6	1.01
Comorbidities								
Diabetes	5 (2.2%)	1,129 (8.8%)	<0.001***	12.80	4 (2.1%)	6 (3.1%)	0.7	0.41
Hypertension	17 (7.3%)	2,477 (19%)	<0.001***	21.44	15 (7.7%)	25 (13%)	0.13	2.79
Smoking	15 (6.5%)	2,758 (22%)	<0.001***	31.13	15 (7.7%)	17 (8.8%)	0.9	0.14
Alcohol use disorder	3 (1.3%)	576 (4.5%)	0.028*	5.55	2 (1.0%)	3 (1.5%)	>0.9	0.20
Substance use disorder	6 (2.6%)	883 (6.9%)	0.014*	6.71	5 (2.6%)	8 (4.1%)	0.6	0.72
COPD	1 (0.4%)	509 (4.0%)	0.009**	7.65	1 (0.5%)	0 (0%)	>0.9	1.00
Hospital characteristics								
Teaching hospital								
Community	161 (69%)	4,570 (36%)	<0.001***	116.21	123 (63%)	128 (66%)	0.8	0.82
Non-teaching	22 (9.5%)	2,331 (18%)			22 (11%)	23 (12%)		
University	45 (19%)	5,757 (45%)			45 (23%)	38 (20%)		
Unknown	4 (1.7%)	106 (0.8%)			4 (2.1%)	5 (2.6%)		
Hospital ownership								
For-profit	10 (4.3%)	1,166 (9.1%)	0.011*	8.96	10 (5.2%)	14 (7.2%)	0.7	0.85
Non-profit/Government	218 (94%)	11,501 (90%)			180 (93%)	175 (90%)		
Unknown	4 (1.7%)	97 (0.8%)			4 (2.1%)	5 (2.6%)		
Hospital size								
Large tertiary centers	28 (12%)	3,324 (26%)	<0.001***	433.3	28 (14%)	26 (13%)	>0.9	0.39
Major academic/Urban hubs	33 (14%)	4,300 (34%)			33 (17%)	34 (18%)		
Medium regional centers	56 (24%)	3,993 (31%)			56 (29%)	52 (27%)		
Small community/Rural centers	115 (50%)	1,147 (9.0%)			77 (40%)	82 (42%)		
Trauma center level								
Level 1	50 (22%)	5,250 (41%)	<0.001***	380.01	50 (26%)	50 (26%)	>0.9	0.07
Level 2	33 (14%)	3,057 (24%)			33 (17%)	34 (18%)		
Level 3	102 (44%)	1,011 (7.9%)			64 (33%)	65 (34%)		
Other	47 (20%)	3,446 (27%)			47 (24%)	45 (23%)		

Fracture characteristics differed between groups. Compared to non-skiers, skiers more frequently sustained closed fractures (88% vs 66%, p < 0.001), displaced fractures (79% vs 68%, p = 0.001), and oblique, spiral, and transverse fracture patterns (13% vs 9.6%; 18% vs 10%; 9.9% vs 8.3%, respectively; p < 0.001). Operative treatment appeared similar between skiers and non-skiers; however, due to inconsistent reporting within the NTDB, this variable was not included in formal comparative analyses (Table 1).

In the unmatched cohort, the non-skier cohort demonstrated longer hospital and intensive care unit (ICU) LOS compared with the skier cohort. Mean hospital LOS was 4 ± 7 days for skiers and 7 ± 9 days for non-skiers. Among patients admitted to the ICU, ICU LOS was significantly longer for non-skiers, with skiers having a mean ICU stay of 2.68 ± 3 days compared with 6.03 ± 8.62 days for non-skiers. In addition, discharge disposition differed significantly between groups: skiers were more frequently discharged home (88% vs 53%, p < 0.001) and less frequently required home health services (3.4% vs 10%, p = 0.001) compared with non-skiers. After propensity matching, these differences in LOS persisted but were attenuated. Complication rates were low in both the unmatched and matched cohorts and did not differ significantly between groups (Table 2). 

**Table 2:** Unmatched and propensity-matched hospital complications and outcomes in the skier and non-skier cohorts. Comparison of complications, discharge disposition, ICU LOS, and hospital LOS between skiing and non-skiing cohorts in unmatched and propensity score-matched samples. Continuous variables are presented as mean ± SD and categorical variables as number (%). BSI, bloodstream infection; SSI, surgical site infection; UTI, urinary tract infection; OR, operating room; CVA, cerebrovascular accident; ICU, intensive care unit; VAP, ventilator-associated pneumonia; ED, emergency department; ICF, intermediate care facility; AMA, against medical advice; SNF, skilled nursing facility; LOS, length of stay; SD, standard deviation. ^a^n (%); Mean ± SD. ^b^Pearson's chi-squared test; NA; Welch two-sample t-test. ^c^NA; Pearson's chi-squared test; Welch two-sample t-test. *p < 0.05; ***p < 0.001.

Characteristic	Unmatched				Matched			
	Skier^a^	Non-skier^a^	p-Value^b^	Statistic	Skier^a^	Non-skier^a^	p-Value^c^	Statistic
Complications								
Central line-associated BSI	0 (0%)	4 (<0.1%)	>0.999	6.14	0 (0%)	0 (0%)	N/A	N/A
Deep SSI	0 (0%)	33 (0.3%)	0.907	0.01	0 (0%)	0 (0%)	N/A	N/A
Deep vein thrombosis	0 (0%)	106 (0.8%)	0.305	1.05	0 (0%)	1 (0.5%)	>0.999	0
Alcohol withdrawal	0 (0%)	57 (0.4%)	0.604	0.27	0 (0%)	0 (0%)	N/A	N/A
Cardiac arrest	0 (0%)	77 (0.6%)	0.450	0.57	0 (0%)	0 (0%)	N/A	N/A
Catheter-associated UTI	0 (0%)	22 (0.2%)	>0.999	8.89	0 (0%)	0 (0%)	N/A	N/A
Pulmonary embolism	1 (0.4%)	63 (0.5%)	>0.999	1.21	1 (0.5%)	0 (0%)	>0.999	0
Extremity compartment syndrome	1 (0.4%)	78 (0.6%)	>0.999	3.72	0 (0%)	2 (1.0%)	0.478	0.50
Intubation	0 (0%)	88 (0.7%)	0.387	0.75	0 (0%)	0 (0%)	N/A	N/A
Acute kidney injury	0 (0%)	70 (0.5%)	0.497	0.46	0 (0%)	0 (0%)	N/A	N/A
Myocardial infarction	0 (0%)	15 (0.1%)	>0.999	1.57	0 (0%)	0 (0%)	N/A	N/A
Organ-space SSI	0 (0%)	10 (<0.1%)	>0.999	6.50	0 (0%)	0 (0%)	N/A	N/A
Osteomyelitis	0 (0%)	8 (<0.1%)	>0.999	1.93	0 (0%)	0 (0%)	N/A	N/A
Other complication	3 (1.3%)	652 (5.1%)	0.013*	6.16	3 (1.5%)	13 (6.7%)	0.022*	5.28
Respiratory	0 (0%)	51 (0.4%)	0.664	0.19	0 (0%)	0 (0%)	N/A	N/A
Unplanned return to OR	0 (0%)	79 (0.6%)	0.438	0.60	0 (0%)	0 (0%)	N/A	N/A
Sepsis	0 (0%)	39 (0.3%)	0.812	0.06	0 (0%)	0 (0%)	N/A	N/A
Stroke/CVA	0 (0%)	32 (0.3%)	0.924	0.009	0 (0%)	1 (0.5%)	>0.999	0
Superficial incision SSI	0 (0%)	24 (0.2%)	>0.999	2.13	0 (0%)	0 (0%)	N/A	N/A
Pressure ulcer	0 (0%)	60 (0.5%)	0.577	0.31	0 (0%)	0 (0%)	N/A	N/A
Unplanned ICU admission	0 (0%)	151 (1.2%)	0.175	1.84	0 (0%)	0 (0%)	N/A	N/A
VAP pneumonia	0 (0%)	63 (0.5%)	0.551	0.35	0 (0%)	0 (0%)	N/A	N/A
Died in ED	0 (0%)	112 (0.8%)	N/A	N/A	0 (0%)	0 (0%)	N/A	N/A
Discharge disposition								
Short-term hospital	0 (0%)	241 (1.9%)	0.062	3.49	0 (0%)	2 (1.0%)	0.478	0.50
ICF	0 (0%)	45 (0.4%)	0.732	0.12	0 (0%)	1 (0.5%)	>0.999	0
Home health	8 (3.4%)	1,288 (10%)	0.001***	10.47	8 (4.1%)	27 (14%)	0.001***	10.17
AMA	0 (0%)	58 (0.5%)	0.595	0.28	0 (0%)	0 (0%)	N/A	N/A
Deceased	0 (0%)	247 (1.9%)	0.058	3.60	0 (0%)	1 (0.5%)	>0.999	0
Home	205 (88%)	6,703 (53%)	<0.001***	116.15	168 (87%)	120 (62%)	<0.001***	29.76
SNF	7 (3.0%)	1,445 (11%)	<0.001***	15.01	6 (3.1%)	14 (7.2%)	0.108	2.58
Hospice	0 (0%)	18 (0.1%)	>0.999	<0.001	0 (0%)	0 (0%)	N/A	N/A
Court/Law enforcement	0 (0%)	0 (0%)	N/A	N/A	0 (0%)	0 (0%)	N/A	N/A
Rehab	0 (0%)	56 (0.4%)	0.613	0.26	0 (0%)	1 (0.5%)	>0.999	0
Long-term care hospital	3 (1.3%)	1,538 (12%)	<0.001***	24.21	3 (1.5%)	5 (2.6%)	0.721	0.127
Psychiatric	0 (0%)	100 (0.8%)	0.330	0.95	0 (0%)	0 (0%)	N/A	N/A
Other institution	0 (0%)	43 (0.3%)	0.758	0.10	0 (0%)	0 (0%)	N/A	N/A
LOS								
ICU LOS (days)	2.68 ± 3	6.03 ± 8.62	<0.001***	-11.49	2.37 ± 2.23	3.22 ± 4.04	0.012*	-0.9
Hospital LOS (days)	4 ± 3	7 ± 9	<0.001***	-15.94	3.47 ± 2.19	4.28 ± 4.00	0.015*	-2.45

In Cox Models 1-3, skiing MOI was consistently associated with a higher hazard of discharge. Specifically, it was associated with increased discharge likelihood after adjustment for demographics and injury severity (HR 1.40, p < 0.001), fracture characteristics (HR 1.77, p < 0.001), and payment and hospital-level factors (HR 1.48, p < 0.001) (Table 3).

**Table 3:** Hazard ratio for skiing status across sequential Cox proportional hazard models for time to hospital discharge. HRs for skiing status compared with other MOIs across four prespecified Cox proportional hazard models. Model 1 adjusted for demographics and injury severity; Model 2 adjusted for fracture characteristics; Model 3 adjusted for payment and hospital factors; and Model 4 incorporated all prior covariates plus comorbidities and ACS status. HRs and 95% CIs were estimated using Cox proportional hazards regression. For each covariate, p‑values correspond to Wald χ² tests with 1 degree of freedom. Hazard ratios greater than 1 indicate a higher hazard of discharge (shorter hospital LOS). HR, hazard ratio; CI, confidence interval; ISS, injury severity score; MOI, mechanism of injury; ACS, acute compartment syndrome; LOS, length of stay. ***p < 0.001.

Model	Exposure	HR	p-Value	Statistic
Model 1: Demographics + ISS	Ski	1.400530	<0.001***	-4.95
Model 2: Fracture characteristics	Ski	1.773159	<0.001***	-8.55
Model 3: Payment + Hospital factors	Ski	1.479533	<0.001***	-5.46
Model 4: Full model	Ski	1.078018	0.302	-1.03

However, in the fully adjusted model (Model 4), skiing MOI was no longer independently associated with time to discharge (HR 1.08, 95% CI 0.93-1.24, p = 0.302) (Tables 3, 4). In this model, factors independently associated with longer LOS included, but were not limited to, increasing age, higher ISS, open fractures, diabetes, smoking, alcohol use disorder, substance use disorder, and ACS (all p ≤ 0.001). Compared with comminuted fractures, spiral, oblique, and transverse fracture patterns were associated with shorter LOS, whereas segmental fractures were associated with longer hospitalization. Hospital characteristics were also independently associated with time to discharge in the fully adjusted model. Compared with large tertiary centers, treatment at small community/rural hospitals was associated with a higher hazard of discharge (HR 1.23, 95% CI 1.14-1.33, p < 0.001), while treatment at major academic/urban centers was associated with a lower hazard of discharge (HR 0.92, 95% CI 0.88-0.97, p = 0.001). Similarly, patients treated at Level III trauma centers had a higher hazard of discharge than those treated at Level I centers (HR 1.20, 95% CI 1.11-1.31, p < 0.001) (Table 4).

**Table 4:** Fully adjusted Cox proportional hazards model for time to hospital discharge (Model 4). Multivariable Cox proportional hazards regression evaluating factors associated with time to hospital discharge among patients with tibial shaft fractures. Model 4 adjusted for demographics, injury severity, fracture characteristics, payment factors, hospital characteristics, comorbidities, and ACS status. HRs and 95% CIs were estimated using Cox proportional hazards regression. For each covariate, p‑values correspond to Wald χ² tests with 1 degree of freedom. The first listed category within each categorical variable served as the reference category in the Cox proportional hazards model. Hazard ratios for the remaining categories are reported relative to this reference category. For binary comorbidity variables, the reference category was the absence of the respective comorbidity. Hazard ratios greater than 1 indicate a higher hazard of discharge (shorter hospital LOS). HR, hazard ratio; CI, confidence interval; ISS, injury severity score; COPD, chronic obstructive pulmonary disease; ACS, acute compartment syndrome; LOS, length of stay. *p < 0.05; ***p < 0.001.

Variable	HR	95% CI	p-Value	Statistic
Ski	1.08	0.93, 1.24	0.3	-1.03
Age	0.99	0.99, 0.99	<0.001***	-22.08
Sex				
Female	--	--	--	--
Male	1.02	0.98, 1.06	0.3	0.94
Race				
Asian	--	--	--	--
Black	1.00	0.86, 1.16	>0.9	-0.02
Other	1.04	0.89, 1.22	0.6	0.53
White	1.14	0.99, 1.32	0.061	1.88
Ethnicity	1.07	1.00, 1.14	0.035*	2.11
Payment type				
Medicare/Medicaid	--	--	--	--
Other	1.10	1.04, 1.16	<0.001***	3.56
Private/Commercial	1.04	0.99, 1.08	0.10	1.66
Fracture type				
Comminuted	--	--	--	--
Oblique	1.25	1.17, 1.33	<0.001***	6.64
Other	1.05	0.95, 1.15	0.3	0.97
Segmental	0.82	0.73, 0.91	<0.001***	-3.77
Spiral	1.42	1.33, 1.52	<0.001***	10.57
Transverse	1.20	1.12, 1.29	<0.001***	5.29
Unspecified	1.14	1.08, 1.19	<0.001***	5.20
Displacement				
Displaced	--	--	--	--
Nondisplaced	1.11	1.02, 1.20	0.015*	2.43
Laterality				
Left	--	--	--	--
Right	1.02	0.99, 1.06	0.2	1.17
Unspecified	1.02	0.93, 1.11	0.7	0.42
Open vs Closed				
Closed	--	--	--	--
Open	0.91	0.88, 0.95	<0.001***	-4.20
ISS	0.95	0.95, 0.95	<0.001***	-37.12
Comorbidities				
Diabetes	0.87	0.81, 0.93	<0.001***	-4.05
Hypertension	0.96	0.91, 1.02	0.2	-1.34
Smoking	0.93	0.88, 0.97	<0.001***	-3.34
Alcoholism	0.85	0.78, 0.93	<0.001***	-3.61
Substance abuse	0.85	0.79, 0.91	<0.001***	-4.43
COPD	0.94	0.85, 1.03	0.2	-1.31
Hospital teaching status				
Community	--	--	--	--
Non-teaching	1.05	0.99, 1.11	0.081	1.74
University	0.95	0.91, 1.00	0.045*	-2.01
Unknown	0.77	0.40, 1.48	0.4	-0.78
Hospital ownership				
For-profit	--	--	--	--
Non-profit/Government	1.07	1.00, 1.14	0.035*	2.11
Unknown	1.48	0.74, 2.96	0.3	1.11
Hospital size				
Large tertiary centers	--	--	--	--
Major academic/Urban hubs	0.92	0.88, 0.97	0.001***	-3.19
Medium regional centers	1.04	0.99, 1.10	0.12	1.58
Small community/Rural centers	1.23	1.14, 1.33	<0.001***	5.38
Hospital trauma level				
Level 1	--	--	--	--
Level 2	1.01	0.95, 1.06	0.8	0.19
Level 3	1.20	1.11, 1.31	<0.001***	4.42
Other	1.15	1.10, 1.21	<0.001***	5.53
ACS	0.41	0.33, 0.50	<0.001***	-8.90

## Discussion

In this national cohort of patients with tibial shaft fractures, skiing-related injuries were associated with shorter hospital LOS and more frequent discharge home in unadjusted and propensity-matched analyses. In the unmatched cohort, skiing patients had shorter mean hospital LOS than non-skiing patients (4 ± 7 vs 7 ± 9 days) and were more frequently discharged home (88% vs 53%, p < 0.001). After propensity matching, the home-discharge difference persisted (87% vs 62%, p < 0.001), although the LOS difference narrowed. In Cox models, skiing MOI was associated with a higher hazard of discharge in partially adjusted analyses, but this association was no longer significant after full adjustment for patient, injury, comorbidity, ACS, and hospital-level factors (HR 1.08, 95% CI 0.93-1.24, p = 0.302). Taken together, these findings indicate that skiing MOI does not independently shorten hospitalization after tibial shaft fractures. Rather, they suggest, but do not establish, that this MOI may identify a distinct lower-risk injury phenotype characterized by younger age, lower injury severity, fewer open fractures, fewer comorbidities, and more favorable discharge disposition.

The discharge disposition findings are central to this interpretation. Skiing patients were substantially more likely to be discharged home and less likely to require home health services, suggesting that this cohort had more favorable short-term recovery and discharge readiness than patients injured through other mechanisms. This aligns with Gitajn et al., who studied 2,365 patients with high-energy lower-extremity trauma and found that 71.5% were discharged home, while older age, higher ISS, severe alcohol abuse, Gustilo IIIB/IIIC open injuries, bilateral injuries, ICU stay, and associated spine or upper-extremity injuries were associated with inpatient-facility discharge [8]. In the present matched cohort, skiing patients had an even higher home-discharge rate than the high-energy lower-extremity cohort reported by Gitajn et al. (87% vs 71.5%), whereas non-skiing patients had a lower home-discharge rate (62% vs 71.5%). This contrast supports the interpretation that skiing tibial shaft fracture patients represent a particularly favorable discharge subgroup rather than a group receiving different care solely because of recreational MOI.

The loss of significance in the fully adjusted model likely reflects the influence of injury severity and comorbidity burden on LOS. These findings are consistent with Lakomkin et al., who studied 1,561 patients with lower-extremity or pelvis fractures and found that patients with a Charlson Comorbidity Index (CCI) score of 1 stayed an average of 7.9 days, whereas patients with a CCI of 3 stayed 1.2 days longer and patients with a CCI of 5 stayed 4.6 days longer; higher CCI scores remained independently associated with longer LOS among lower-extremity fracture patients [9]. In the present study, the unmatched cohort of skiing patients had lower ISS than non-skiing patients (5 ± 2 vs 10 ± 9, p < 0.001), fewer open fractures (12% vs 34%, p < 0.001), and lower rates of diabetes, hypertension, smoking, alcohol use disorder, substance use disorder, and COPD. These findings suggest that the apparent LOS advantage among skiing patients was largely explained by the healthier baseline profile and lower acute injury burden of this cohort.

Fracture morphology and open fracture status further reinforce this interpretation. Basques et al. evaluated 6,055 skiing and snowboarding injuries in the NTDB and found that 55.2% occurred in skiers, 61% were fractures, and lower-extremity fractures were the most common orthopedic injury and occurred more often among skiers than snowboarders [10]. In the present cohort, skiing patients were more likely than non-skiing patients to have closed fractures (88% vs 66%, p < 0.001) and spiral fractures (18% vs 10%). Johner et al. provide a useful mechanism-based comparison: among 210 tibial shaft fractures, 86 indirect-impact injuries were primarily spiral fractures with fewer soft-tissue injuries, whereas 124 direct-impact injuries were more often transverse, oblique, segmental, or crush injuries with greater soft-tissue damage [11]. This pattern corresponds with the present study, in which open fracture status was independently associated with delayed discharge (HR 0.91, 95% CI 0.88-0.95, p < 0.001), spiral fractures were associated with faster discharge than comminuted fractures (HR 1.42, 95% CI 1.33-1.52, p < 0.001), and segmental fractures were associated with delayed discharge (HR 0.82, 95% CI 0.73-0.91, p < 0.001). The contrast with segmental tibia fracture literature is notable: Corey et al. identified 108 segmental tibial shaft fractures, including 73 open fractures (67%), 34 cases of compartment syndrome (31%), a mean ISS of 29, a mean LOS of 13 days, delayed union in 43%, and nonunion in 9% [12]. By comparison, only 1.3% of skiing patients in the present cohort had segmental fractures, 12% had open fractures, the mean ISS was 5 ± 2, and the mean hospital LOS was four days. These comparisons indicate that fracture complexity and soft-tissue injury burden, rather than skiing MOI itself, are key drivers of short-term hospitalization.

Hospital-level findings should be interpreted as markers of case mix and system context rather than evidence that one facility type provides intrinsically faster care. These findings are consistent with Schwartz et al., who studied 28,314 orthopedic polytrauma patients and found that treatment at high-acuity centers was associated with 2.8 days longer LOS, along with higher odds of ICU admission, ventilatory support, unplanned reoperation, and medical complications [13]. This pattern was reflected in our fully adjusted model, in which treatment at small community or rural hospitals was associated with faster discharge than treatment at large tertiary centers (HR 1.23, 95% CI 1.14-1.33, p < 0.001), whereas treatment at major academic or urban hospitals was associated with delayed discharge (HR 0.92, 95% CI 0.88-0.97, p = 0.001). Similarly, Level III trauma centers were associated with faster discharge than Level I centers (HR 1.20, 95% CI 1.11-1.31, p < 0.001). It should be noted that discharge timing may also reflect post-acute placement barriers. Hwabejire et al. found that among trauma patients with excessively prolonged hospitalization, only 20% of discharge delays were clinical, whereas 47% were related to rehabilitation facility placement, 26% to in-hospital operational delays, and 7% to payer-related issues [14]. Overall, skiing patients were more often discharged home, while non-skiing patients more frequently required home health, skilled nursing facilities (SNFs), long-term care hospitals, or other institutional dispositions. Thus, LOS differences likely reflect both clinical injury burden and downstream discharge pathways.

This study has several limitations inherent to retrospective analyses of large national trauma registries. First, the NTDB relies on ICD-10 diagnosis and external-cause coding, which may introduce misclassification of tibial shaft fractures, fracture morphology, open versus closed injury status, skiing MOI, and associated complications. As with all registry-based studies, the accuracy of the data depends on the completeness and correctness of coding at participating institutions, and certain diagnoses, procedures, and complications may have been incompletely captured compared with detailed chart review. Second, fracture morphology coding was incomplete, with fracture pattern classified as unspecified in 16% of skiers and 22% of non-skiers in the unmatched cohort. Third, the database lacks granular clinical information regarding Gustilo-Anderson classification, soft-tissue injury severity, fixation strategy, timing of surgery, weight-bearing status, rehabilitation protocols, transfer status, and discharge decision-making. Fourth, operative treatment was not included in the primary Cox model because procedural coding appeared unreliable; notably, despite open tibial shaft fractures generally representing a strong indication for operative fixation, operative treatment was coded in only approximately half of open fracture cases. Including this variable may therefore have introduced bias from incomplete procedural capture, and sensitivity modeling showed that adding operative treatment did not materially change the association between skiing MOI and time to discharge. Fifth, although propensity score matching and multivariable Cox regression were used to reduce confounding, residual confounding remains possible because unmeasured variables such as baseline mobility, preinjury functional status, social support, caregiver availability, rehabilitation access, transfer patterns, and local discharge practices could not be assessed. Sixth, the skiing cohort was small relative to the non-skiing cohort, which may limit precision for less common outcomes and subgroup analyses. Finally, the NTDB is limited to the index trauma hospitalization, preventing evaluation of emergency department discharges, readmissions, reoperations, fracture union, infection, functional recovery, return to activity, and long-term resource utilization.

## Conclusions

In this national database study of patients with tibial shaft fractures, skiing- and snowboarding-related injuries initially appeared to be associated with shorter hospital LOS, shorter ICU LOS, and higher rates of home discharge compared with injuries sustained through other MOIs. However, this MOI was no longer independently associated with time to discharge after adjustment for patient-, injury-, comorbidity-, and hospital-level characteristics in the fully adjusted Cox model. While this MOI does not appear to independently drive short-term hospital outcomes, these findings suggest, but do not establish, that skiing- and snowboarding-related tibial shaft fractures represent a distinct lower-risk injury profile characterized by younger age, lower injury severity, fewer open fractures, fewer comorbidities, and more favorable discharge disposition. Future studies are needed to definitively establish whether these findings extend to long-term outcomes and whether skiing- and snowboarding-related tibial shaft fractures truly represent a distinct injury phenotype with unique patient, injury, and treatment characteristics.
